# Surgical Outcome of Renal Cell Carcinoma with Tumor Thrombus Extension into Inferior Vena Cava and Right Atrium (Beating Heart Removal of Level 4 Thrombus): A Challenging Scenario

**DOI:** 10.15586/jkcvhl.2020.149

**Published:** 2020-07-31

**Authors:** Abdul Rouf Khawaja, Khalid Sofi, Yasir Dar, Muzaain Khateeb, Javeed Magray, Abdul Waheed, Sajad Malik, Arif Hamid Bhat, Mohd. Saleem Wani, Akbar Bhat

**Affiliations:** 1 Department of Urology, Department of Cardiovascular surgery, Sheri Kashmir Institute of Medical Sciences (SKIMS), Srinagar Soura, Jammu and Kashmir, India; 2 Department of Anaesthesiology, Department of Cardiovascular surgery, Sheri Kashmir Institute of Medical Sciences (SKIMS), Srinagar Soura, Jammu and Kashmir, India

**Keywords:** renal cell carcinoma, tumor thrombus, cardiopulmonary bypass, intraoperative transesophageal echocardiography

## Abstract

**Aim:**

“To evaluate oncological and surgical outcomes of different levels of tumor thrombus and tumor characteristics secondary to renal cell carcinoma (RCC)”.

**Materials and Methods:**

Retrospective review from 2013 to 2020 of 34 patients who underwent radical nephrectomy with thrombectomy for RCC with tumor thrombus extending into the inferior vena cava (IVC) and right atrium (RA) at our center. Level I and most level II tumors were removed using straight forward occluding maneuvers with control of the contralateral renal vein. None of the patients had level III tumor extensions in our study group. For level IV thrombus, a beating heart surgery using a simplified cardiopulmonary bypass (CPB) technique was used for retrieval of thrombus from the right atrium.

**Results:**

“ Of the 34 patients with thrombus”, 19 patients had level I, 12 patients had level II, none had level III, and three patients had level IV thrombus. Two patients required simplified CPB. Another patient with level IV thrombus CPB, was not attempted in view of refractory hypotension intraoperatively. Pathological evaluation showed clear-cell carcinoma in 67.64%, papillary carcinoma in 17.64%, chromophobe in 5.8%, and squamous cell carcinoma in 8.8% of cases. Left side thrombectomy was difficult surgically, whereas right side thrombectomy did not have any survival advantage. Mean blood loss during the procedure was 325 mL, ranging from 200 to 1000 mL, and mean operative time was 185 min, ranging from 215 to 345 min. The immediate postoperative mortality was 2.9%. Level I thrombus had better survival compared to level II thrombus.

**Conclusion:**

Radical nephrectomy with tumor thrombectomy remains the mainstay of treatment in RCC with inferior venacaval extension. The surgical approach and outcome depends on primary tumor size, location, level of thrombus, local invasion of IVC, any hepato-renal dysfunction or any associated comorbidities. The higher the level of thrombus, the greater is the need for prior optimization and the adoption of a multidisciplinary approach for a successful surgical outcome.

## Introduction

Renal cell carcinoma (RCC) is responsible for 2–3% of all adult malignancies and is one of the fatal tumors of the renal system. RCC consists of various subtypes associated with different parts of the nephron, each with specific genetic and tumor biology (1). A striking and significant feature of RCC lies in its tendency to grow intraluminally into the renal venous system in a cranial direction known as venous tumor thrombosis. The growth may extend till the right atrium (RA) or beyond. This growth of RCC into the renal venous system has been observed among 4–10% of the patients. With frequent use of ultrasonography, the diagnosis of RCC with venous involvement has steadily increased even in asymptomatic cases (2). Fortunately, 45–70% of the patients with venous tumor thrombosis can be successfully treated

by nephrectomy combined with thrombectomy. Among patients with a primary diagnosis of renal mass, the presence of lower extremity edema, dilated superficial abdominal veins, proteinuria, pulmonary embolism, isolated right-sided varicocele, and right atrial mass raises the suspicion of renocaval tumor extension. “Surgery becomes more complex; the more the extension of the thrombus, the higher the morbidity and mortality”. A multidisciplinary approach is required for the management of these patients, and it is a challenging scenario for the uro-oncologist.

## Materials and Methods

It was a retrospective study in which all patients with a diagnosis of renal cell carcinoma (RCC) with tumor thrombus extension into the inferior vena cava (IVC) and right atrium (RA) were enrolled. These patients had been admitted and operated upon at our center, the Sher-i-Kashmir Institute of Medical Sciences (SKIMS), from January 2013 to January 2020. Patients who had received neoadjuvant targeted therapy were excluded from the study. In all patients, Contrast-Enhanced Computed Topography/Magnetic Resonance imaging (CECT/MRI) was performed to assess the cephalic extension of the thrombus. Chest X-ray and bone scans were performed preoperatively in all patients to exclude metastasis. The level of renocaval thrombus was staged as follows: level I: adjacent to the ostium of the renal vein; level II: extending up to the lower aspect of the liver; level III: involving the intrahepatic portion of the IVC but below the diaphragm; and level IV: extending above the diaphragm. Based on surgeon preference and the level of thrombus, the surgical approach was selected. Level 1 and most level II thrombi were removed using straight forward occluding maneuvers with control of the contralateral renal vein for right-sided tumors and control of right renal artery for left-sided tumors. For level IV thrombi, a beating heart surgery with a simplified cardiopulmonary bypass (CPB) technique was used for the retrieval of the thrombus from RA.

## Operative Technique for Different Levels of Thrombus

**Figure 1:**
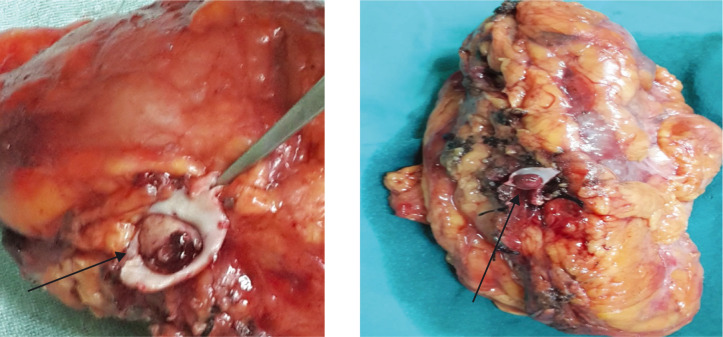
Level 1 thrombus (HPE Squamous cell carcinoma) with arrows showing level 1 thrombus.

**Figure 2:**
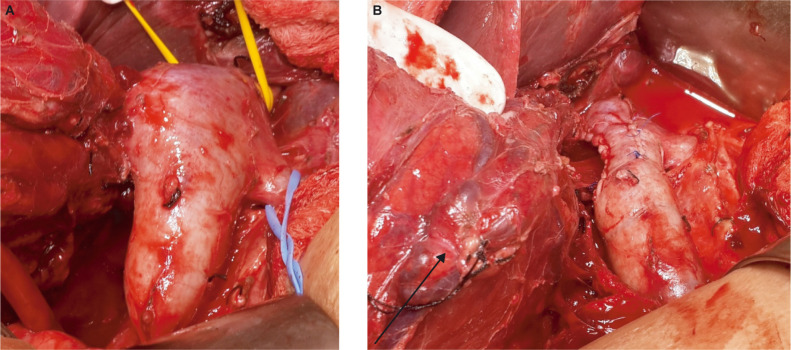
(A) Level 2 thrombus with proximal, distal and left renal tourniquet. (B) Completion of tumor thrombectomy with renal mass (arrow) in situ.

**Figure 3:**
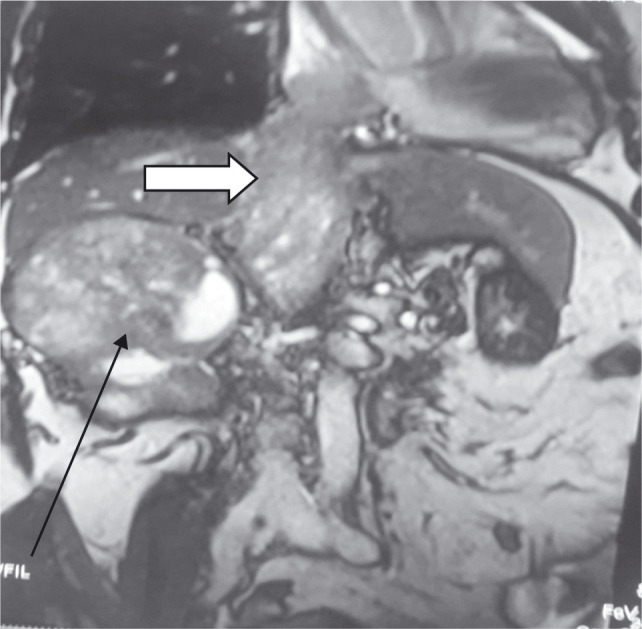
Level 4 thrombus with arrow (Black) showing mass in right kidney and white arrow thrombus in IVC extending to right atrium.

**Figure 4:**
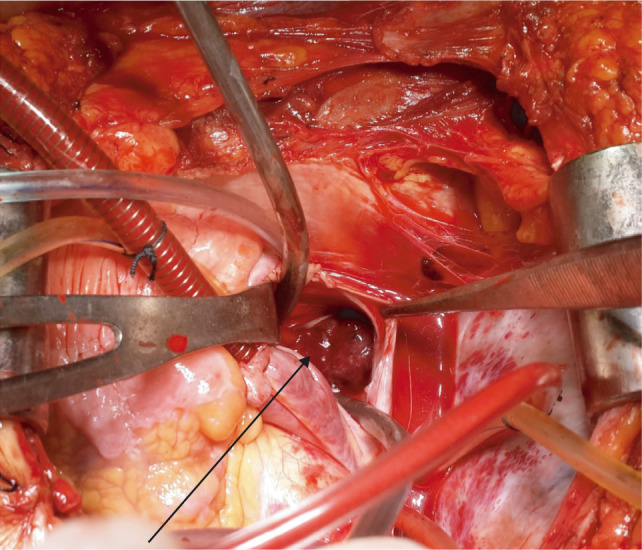
Opening of right atrium with arrow showing thrombus in situ.

The surgical technique used for different levels of thrombus extension in our study is as follows. For level I (Figure 1) thrombus, vascular side clamps are used without occluding IVC inflow. The cavotomy defect is then sewn using a continuous prolene 4-0 suture. Level II thrombus (Figure 2A and B) often necessitates more extensive mobilization of the IVC. Proximal and distal control of IVC is necessary with ligation and division of lumbar veins to prevent significant blood loss. For the retrieval of thrombus, vascular clamps are used with clamping of the left renal vein in case of right-sided renal tumors and clamping of the right renal artery in case of left-sided renal tumors. The cavotomy defect is sewn using continuous prolene 4-0 suture. For level IV thrombi (Figure 3), a beating heart surgery with a simplified CPB technique was used for retrieval of thrombus from RA. For level IV thrombi, ligation of renal artery is followed by radical nephrectomy using chevron incision. The left renal vein is looped, and IVC below the renal vein is skeletonized for a length of about 5 cm to prepare it for IVC cannulation. A purse string is made on it using 4-0 prolene monofilament suture and the subhepatic portion of IVC is dissected. Then, median sternotomy is performed and pericardium is opened. Aortic purse-string suture with 3-0 prolene is taken on the ascending aorta. The intrapericardial portion of the superior vena cava (SVC) is looped, and a purse-string with 3-0 prolene is taken. Patient is heparinized with 300 Units/kg body weight of heparin. Aortic and venous cannulations are performed, and patient is put on complete CPB after both SVC and IVC are snugged with Rummel tourniquet. No vent cannula is put in. Patient is cooled to 35°C. On beating heart under normothermic CPB, RA thrombus (Figure 4) is extracted without cross-clamping the aorta. Right atriotomy is performed and tumor thrombus, which is organized and firm, is carefully retrieved. IVC is snugged at its junction with RA to prevent spillover from IVC into RA. Subhepatic IVC is incised longitudinally, and the tumor thrombus is extracted, using digital manipulation to ensure complete removal from retrohepatic IVC. Intermittent suctioning is performed using a pump sucker as hepatic veins are opened up. Cavotomy is closed using 4-0 prolene suture. After completely de-airing the RA, the atriotomy is also closed. Patient is gradually weaned off the bypass. “Patients were followed up quarterly in the first year, semiannually in the second year, and annually thereafter”.

## Results

A total of 34 patients who had been diagnosed with RCC with venous tumor extension over a period of 7 years were enrolled in this study. All these patients underwent radical nephrectomy with thrombectomy at our center as per the surgical techniques detailed above, depending on the level of tumor extension. The mean age of the study group was 66 ± 8 years, with the youngest patient being 17 years old and the oldest being 71 years old. The group included 24 male and 10 female patients with a male:female ratio of 2.4:1. Of the 34 patients, 27 patients (79.42%) had right kidney tumor while seven patients (20.58%) had left kidney involvement. Among the 34 patients, 19 (55.9%) had level I thrombus, 12 (35.3%) had level II thrombus, and three (8.8%) had level IV tumor thrombus extension. None of the patients in our study group had level III tumor extension.

Of the 34 patients, 28 patients had no intraoperative and major postoperative complications. The details of remaining 6 patients are shown in Table 1. Mean blood loss during the procedure was 325 mL, ranging from 200 to 1000 mL, and mean operative time was 185 min, ranging from 215 to 345 min. One patient (2.9%) with level IV thrombus died immediately after surgery. Histopathological examination revealed clear-cell carcinoma in 23 cases (67.64%), papillary carcinoma in six cases (17.64%), chromophobe in two cases (5.8%), and squamous cell carcinoma in 3 cases (8.8%). Mean follow-up of patients in our study group was 7 years ± 3 months. “Level I thrombus patients had better survival when compared to level II thrombus patients”.

**Table 1:** Clinical profile of patients (N = 34)

Male Female	24 10
Laterality Right Left	27 7
Tumor Thrombus level Level 1 Level 2 Level 3 Level 4	19 12 0 03
Histopathological subtype of renal cell carcinoma Clear cell carcinoma Papillary cell carcinoma Chromophobe cell carcinoma Squamous cell carcinoma	67.64% 17.64% 5.8% 8.8%
Complications Peropeartive death Deep vein thrombosis Wound infection	01 03 07

## Discussion

**Figure 5:**
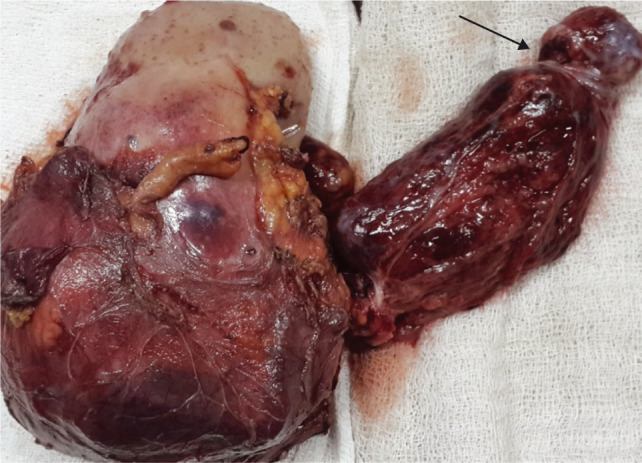
Completion of nephrectomy with tumor thrombus level 4 with arrow showing thrombus removed from atrium and IVC.

Approximately, 2–16% of RCC patients had extension of tumor thrombus to RA (4–6). In the 2010 International Union against Cancer TNM staging system, the diameter of renal tumors and the level of tumor thrombus are important prognostic indicators. This surgical procedure has two components for the excision of renal mass and IVC tumor thrombus. In our series, majority of patients had level I thrombus (55.88%), and histopathological examination of resected specimens (19 nos.) revealed squamous cell carcinoma in 03 cases (15.78%), clear-cell carcinoma in ten cases (52.63%), papillary carcinoma in 04 cases (21.05%), and chromophobe cell carcinoma in 02 case (10.52%). In this cohort of level I thrombus, 16 patients were alive till last follow-up until 5 years with no recurrence of tumor thrombus or distant metastasis. Three patients with histopathological examination HPE of squamous cell carcinoma died within 2 years of surgery and one patient developed lung metastasis. Of the 12 patients (35.29%) with level II thrombus, 10 (83.34%) had clear-cell carcinoma and two (16.66%) had papillary carcinoma. In the level IV thrombus group (8.8%), histopathological examination revealed clear cell carcinoma with sarcomatoid variant in all three cases and only two were completely resected. In one case only biopsy was taken in view of intraoperative hemodynamic instability. All the resected specimens had a negative margin. One patient with left RCC developed solitary lung metastasis during-follow up and was put on tyrosine kinase inhibitors (TKIs) by the medical oncologist. The patient behaved well but was lost to follow-up after 3 years. The more the length of the tumor thrombus, the more challenging it is, necessitating a multidisciplinary approach that increases the complexity of the management. “Radical resection is the only curative option for RCC with a tumor thrombus in the inferior vena cava, and accurate pre operative imaging is crucial for a positive surgical outcome”. Most patients with IVC extension can be treated with nephrectomy with IVC thrombectomy without putting the patient on CPB; however, in cases of RA extension (level IV), CPB is strongly recommended (5). Digital manipulation of tumor thrombus to push it from RA into IVC without CPB can cause life-threatening blood loss. CPB offers a bloodless field and hemodynamic stability during thrombectomy from IVC and RA (2, 7–9). There are, however, inherent complications associated with CPB, including increase in operative time, myocardial ischemia during cross-clamp, arrhythmias, respiratory complications, and postoperative hemorrhagic drainage due to onset of coagulopathies (5, 10). In literature, some surgeons have used profound hypothermia with circulatory arrest to retrieve the tumor thrombus from RA (11, 12). The predominant limitations of this technique are its associated morbidities, including neurologic complications (delirium, psychosis, and stroke) (13, 14), thrombocytopenia, and platelet dysfunction, resulting in significant bleeding, prolonged and/or improper rewarming with postoperative hypothermia, and overall higher mortality (13, 15). Our approach in level IV thrombus (Figure 5) was beating heart on simplified CPB without cross-clamp on aorta. Beating heart is more physiological, as the heart is allowed to beat normally, maintaining a normal coronary perfusion and contractility, while offloading the heart of blood volume. As a result, this allows for smooth resumption of heart off bypass. Weaning off from CPB is quick, smooth, and easy, without having to wait for the mandatory period to replenish the myocardium with energy substrates needed in the case of an arrested heart. This reduces the operative time, and facilitates better hemodynamic stability, early extubation, and early shifting from intensive care unit to the general ward. As a result of shorter CPB time, the inherent risks of extracorporeal circulation are minimized, although not eliminated. These include less systemic inflammatory response, less coagulopathy and thrombocytopenia, thereby less postoperative bleeding and blood transfusions; less chances of embolism, stroke, and post-CPB psychosis.

The disadvantages of this approach include: chances of intraoperative bleeding from cavotomy or arteriotomy are higher in beating heart, and it negatively impacts visibility, if tumor thrombus has extended to RA or RV across the tricuspid valve. Arrested heart has a definite advantage in this regard. Risk of paradoxical air or tumor embolism via Atrial septal defect/Patent foramen ovale (ASD or PFO) to brain, coronaries, or other organs can have catastrophic results; however, the chances for such adverse events are minimal given the on-table maneuvers like head down, and the avoidance of the emptying of left atrium.

Blood loss is minimal in circulatory arrest due to the standstill circulatory system at profound hypothermia. But in the beating heart technique, the blood loss, which happens due to hepatic venous and lumbar venous return, is collected using a pump sucker via the inferior cavotomy and returned back to pump recirculation, and this is what we did in two cases and blood loss was minimal. In occasional cases, where the cavotomy blood loss may overwhelm the pump suction capacity, a brief Pringle maneuver, at the cost of some hepatic ischemia, can be employed to stop hepatic venous return till the thrombus is retrieved and the cavotomy is closed. However, we did not required to adopt this maneuver in both the cases. Predominant advantages of this approach include shorter bypass time and operative time, as no time was required for rewarming, more physiological for myocardium due to continuous coronary perfusion, bloodless field, except for bleeding from hepatic veins which was taken care of by pump suckers, less coagulopathy and postoperative bleeding. The morbidity related to hypothermic circulatory arrest and aortic cross-clamping was avoided. Thus, our approach was easy to set up, safe, and simple to use. It is ideal to have intraoperative Transesophageal echocardiography (TEE), a noninvasive technique, which is very helpful in confirming the exact position of the thrombus within IVC and RA, and ensures complete thrombectomy (16, 17). In our series, a total of three patients with level IV thrombus were included. Two patients had a smooth intraoperative course and the final biopsy revealed a clear-cell carcinoma with a sarcomatoid variant. Both the patients were disease-free for first 2 years of follow-up; one patient died in another hospital due to acute myocardial infarction, while another patient developed recurrence at the adrenal region and was put on TKIs (sunitinib) by medical oncology and subsequently died after detection of recurrence at 6 months. One patient with level IV thrombus had intraoperative hemodynamic instability on opening the abdomen, as significant amount of ascitic fluid (approximately 2 L) was drained, to achieve early control of the right renal artery. All pressor supports were tried; however, on the operating table, the patient had refractory hypotension, and the cardiac surgeon refused to put the patient on cardiopulmonary bypass because of persistent hypotension. Only renal biopsy was performed. Patient was intubated to surgical intensive care unit (SICU) for further management. As patient had preoperatively hepatic dysfunction because of hepatic congestion but her International normalized ratio (INR) was 1.4, with raised creatinine 1.9 mg dL. Patient developed azotemia (acute kidney injury) in the SICU and was put on dialysis by the nephrology team, but the patient continued to be sick and ultimately died in the SICU without undergoing nephrectomy and thrombectomy. Retrospectively, the fatality could be the result of the sudden withdrawal of the ascitic fluid without any cover of albumin, or the rapid reduction of atrial pressure, or the vasodilation in splanchnic bed with reduced venous return to the heart as IVC was completely occluded by tumor thrombus. Another explanation was the poorly developed collaterals. The entire multidisciplinary team believed that the patient’s refractory hypotension was the result of aggressive removal of ascitic fluid and poorly developed collaterals. To avoid this hemodynamic instability on the operating table, we believe that such patients need serial paracentesis before surgery under albumin coverage. Such a complication has not been reported in literature, and further study for such initial intraoperative hemodynamic instability is necessary. We believe that the clinical and operative characteristics of different levels of reno-caval tumor thrombus have a significant impact on the surgical outcome. We have not used any neoadjuvant systemic therapy in any of our cases. The rationale for not using targeted therapy before surgery as upfront surgical resection remains the cornerstone of any RCC. Preoperatively targeted therapy with TKIs is hypothesized to facilitate resectability, reduce surgical morbidity, and allow for nephron-sparing surgery. In our case series, none of the patients had bilateral tumors or tumor in a solitary kidney with IVC tumor thrombus. Moreover, the duration and timing of TKI is varied among the previous reports, and the associated toxicity and cost of the treatment is an impediment in its use in level IV thrombus.

## Conclusion

Radical nephrectomy with tumor thrombectomy remains the mainstay of treatment in RCC with IVC extension. The surgical approach and outcome depends on primary tumor size, location, level of thrombus, local invasion of IVC, any hepatorenal dysfunction, or other associated comorbid conditions. The higher the level of the thrombus, the more is the need for prior optimization and a multidisciplinary approach for a successful surgical outcome.

## References

[1] Rini BI, Wilding G, Hudges G, Stadler WM, Kim S, Tarazi J, et al Phase II study of axitinib in sorafenib-refractory metastatic renal cell carcinoma. J clinical Oncol. 2009;27(27):4462–8. 10.1200/JCO.2008.21.7034 19652060

[2] Novick AC, Kaye MC, Cosgrove DM, Angermeier K, Pontes JE, Montie JE, et al Experience with cardiopulmonary bypass and deep hypothermic circulatory arrest in the management of retroperitoneal tumors with large vena caval thrombi. Ann Surg. 1990;212(4):472–6. 10.1097/00000658-199010000-00010 2222013 PMC1358282

[3] Gastro GJ, Mckiernan JM Epidemiology, clinical staging, and presentation of renal cell carcinoma Urol Clin North America 2008 35(4):581–92. 10.1016/j.ucl.2008.07-005. 18992612

[4] Skinner DG, Pritchett TR, Lieskovsky G, Boyd SD, Stiles QR Vena caval involvement by renal cell carcinoma. Surgical resection provides meaningful long-term survival. Ann Surg. 1989;210(3):387–92. 10.1097/00000658-198909000-00014 PMC1358008 2774709

[5] Nesbitt JC, Soltero ER, Walsh GL, Schrump DS, Swanson DA, Pisters LL, et al Surgical management of renal cell carcinoma with inferior vena cava tumor thrombus. Ann Thorac Surg. 1997;63(6):1592–600. 10.1016/S0003-4975(97)00329-9 9205155

[6] Neves RJ, Zincke H Surgical treatment of renal cancer with vena cava extension. Br J Urol. 1987;59(5):390–5. 10.1111/j.1464-410X.1987.tb04832.x 3594097

[7] Glazer AA, Novick AC Long-term followup after surgical treatment for renal cell carcinoma extending into the right atrium. J Urol. 1996;155(2):448–50. 10.1016/S0022-5347(01)66415-2 8558632

[8] Yamashita C, Ataka K, Azami T, Nakagiri K, Wakiyama H, Okada M Usefulness of cardiopulmonary bypass in reconstruction of inferior vena cava occupied by renal cell carcinoma tumor thrombAngiology. 1999;50(1):47–53. 10.1177/000331979905000106 9924888

[9] Montie JE, el Ammar R, Pontes JE, Medendorp SV, Novick AC, Streem SB, et al Renal cell carcinoma with inferior vena cava tumor thrombi. Surg Gynecol Obstet. 1991;173(2):107–15. 1925859

[10] Gallucci M, Borzomati D, Flammia G, Alcini A, Albino G, Caricato M, et al Liver harvesting surgical technique for the treatment of retro-hepatic caval thrombosis concomitant to renal cell carcinoma: Perioperative and long-term results in 15 patients without mortality. Eur Urol. 2004;45(2):194–202. 10.1016/j.eururo.2003.09.004 14734006

[11] Marshall FF, Reitz BA, Diamond DA A new technique for management of renal cell carcinoma involving the right atrium: Hypothermia and cardiac arrest. J Urol. 1984;131(1):103–7. 10.1016/S0022-5347(17)50221-9 6690724

[12] Krane RJ, de Vere White R, Davis Z, Sterling R, Dobnik DB, McCormick JR Removal of renal cell carcinoma extending into the right atrium using cardiopulmonary bypass, profound hypothermia and circulatory arrest. J Urol. 1984;131(5):945–7. 10.1016/S0022-5347(17)50722-3 6708232

[13] Okita Y, Takamoto S, Ando M, Morota T, Matsukawa R, Kawashima Y Mortality and cerebral outcome in patients who underwent aortic arch operations using deep hypothermic circulatory arrest with retrograde cerebral perfusion: No relation of early death, stroke, and delirium to the duration of circulatory arrest. J Thorac Cardiovasc Surg. 1998;115(1):129–38. 10.1016/S0022-5223(98)70451-9 9451056

[14] Ergin MA, Galla JD, Lansman L, Quintana C, Bodian C, Griepp RB Hypothermic circulatory arrest in operations on the thoracic aorta. Determinants of operative mortality and neurologic outcomThorac Cardiovasc Surg. 1994;107(3):788–97. 10.1016/S0022-5223(94)70334-5 8127108

[15] Svensson LG, Crawford ES, Hess KR, Coselli JS, Raskin S, Shenaq SA, et al Deep hypothermia with circulatory arrest. Determinants of stroke and early mortality in 656 patients. J Thoraccardiovasc Surg. 1993;106(1):19–28. 8321002

[16] Glazer A, Novick AC Preoperative transesophageal echocardiography for assessment of vena caval tumor thrombi: A comparative study with venacavography and magnetic resonance imaging. Urology. 1997;49(1):32–4. 10.1016/S0090-4295(96)00374-3 9000181

[17] Sigman DB, Hasnain JU, Del Pizzo JJ, Sklar GN Real-time transesophageal echocardiography for intraoperative surveillance of patients with renal cell carcinoma and vena caval extension undergoing radical nephrectomy. J Urol. 1999;161(1):36–8. 10.1016/S0022-5347(01)62054-8 10037362

